# Long-term misdiagnosis of Niemann–Pick disease type C as Wilson disease: A case report

**DOI:** 10.1097/MD.0000000000049790

**Published:** 2026-07-10

**Authors:** Shang Xiang, Daiping Hua, Lijuan Zhang, Lanting Sun, Wenming Yang, Han Wang

**Affiliations:** a Department of Neurology, The First Affiliated Hospital of Anhui University of Chinese Medicine, Hefei, China; b Key Laboratory of Xin’an Medicine, Ministry of Education, Hefei, China.

**Keywords:** genetic testing, Leipzig scoring system, misdiagnosis, Niemann–, Pick disease type C, Wilson disease

## Abstract

**Rationale:**

Niemann–Pick disease type C (NPC) is a rare autosomal recessive lysosomal lipid storage disorder caused by pathogenic variants in *NPC1* or *NPC2*. Adult-onset NPC may manifest with progressive ataxia, dysarthria, cognitive impairment, psychiatric symptoms, and splenomegaly, features that may overlap with Wilson disease (WD). Abnormal copper metabolism in NPC can further complicate the differential diagnosis.

**Patient concerns:**

A 39-year-old man was admitted with a more than 15-year history of slowly progressive dysarthria and gait instability, accompanied by intermittent choking when drinking and mild recent memory impairment. He had previously been diagnosed with WD because of splenomegaly, low serum ceruloplasmin, and increased 24-hour urinary copper excretion, and had received intermittent copper-chelating therapy. His 42-year-old sister had similar neurological symptoms and had also been diagnosed with WD.

**Diagnoses:**

On readmission, the patient had leukopenia, splenomegaly, low serum ceruloplasmin, and elevated 24-hour urinary copper excretion. His Leipzig score was 4, meeting the diagnostic threshold for WD. However, Kayser–Fleischer rings were absent, brain magnetic resonance imaging showed no typical abnormalities suggestive of neurological WD, and the disease course was unusually indolent despite long-term irregular anti-copper treatment. Genetic testing of the proband and his affected sister identified compound heterozygous pathogenic variants in *NPC1*, c.2932C > T (p.Arg978Cys) and c.2974G > T (p.Gly992Trp), confirming the diagnosis of NPC.

**Interventions:**

Copper-chelating therapy was discontinued after the diagnosis was revised. The patient was started on miglustat, initially at 0.2 g once daily, which was gradually increased to 0.2 g 3 times daily.

**Outcomes:**

After 3 months of follow-up, the patient’s dysphagia improved, whereas gait instability persisted.

**Lessons:**

Adult-onset NPC can mimic WD clinically and biochemically. Because copper metabolism abnormalities are not pathognomonic for WD, they may yield misleadingly high Leipzig scores. NPC should be prioritized in patients with progressive neurological decline and splenomegaly who lack classic WD hallmarks, such as Kayser–Fleischer rings or characteristic neuroimaging findings. Early genetic testing is essential to ensure diagnostic accuracy and avoid inappropriate long-term copper-chelating therapy.

## 1. Introduction

Niemann–Pick disease type C (NPC) is a rare autosomal recessive lysosomal lipid storage disorder. The clinical manifestations of adolescent- and adult-onset NPC are complex, frequently presenting with progressive ataxia, dysarthria, tremor, cognitive decline, and psychiatric abnormalities as primary symptoms, occasionally accompanied by hepatosplenomegaly. Consequently, it is easily confused with Wilson disease (WD).^[1]^ WD is a copper metabolism disorder caused by genetic variants, and the Leipzig scoring system currently serves as a crucial tool for its clinical diagnosis.^[2]^ However, scoring indicators such as decreased ceruloplasmin, elevated urinary copper, and neurological symptoms are not specific to WD; similar abnormalities can also occur in other inherited metabolic diseases like NPC, potentially leading to misdiagnosis and inappropriate long-term copper-chelating therapy. This study reports a patient who was misdiagnosed with WD for an extended period and ultimately diagnosed with NPC via genetic testing. By synthesizing the patient’s clinical features and diagnostic trajectory, we analyze the key differential points between NPC and WD and explore the limitations of the Leipzig scoring system in complex cases to improve clinical recognition of NPC.

## 2. Case presentations

A 39-year-old man presented with a more than 15-year history of slowly progressive dysarthria and gait instability. The disease onset was insidious, with slurred speech, intermittent choking when drinking liquids, gait unsteadiness, and mild impairment of recent memory. He had previously been evaluated at another hospital, where splenomegaly, a low serum ceruloplasmin level of 0.109 g/L (normal reference range: 0.23–0.44 g/L), and elevated 24-hour urinary copper excretion of 620 µg/24 h (normal reference range: <100 µg/24 h) led to a diagnosis of WD. The patient was first admitted to our center in August 2017 with a presumptive diagnosis of WD. Abdominal ultrasonography confirmed splenomegaly, and serum ceruloplasmin remained low at 0.136 g/L. He was treated with hepatoprotective agents and copper-chelating therapy, resulting in partial symptomatic improvement. After discharge, however, he was lost to regular follow-up and did not consistently receive anti-copper treatment from 2017 to 2025. During this period, his neurological manifestations progressed only minimally, and he remained largely independent in daily life. In late 2025, he noted worsening dysarthria and began taking penicillamine on his own. By early 2026, his dysarthria and gait instability had further deteriorated, accompanied by increased choking when drinking liquids and the emergence of sphincter dysfunction. He was readmitted to our hospital on March 9, 2026, for reassessment. He had no other relevant medical history. Growth and developmental milestones were normal. He was unmarried and was born to non-consanguineous parents. His 42-year-old sister had a 9-year history of progressive dysarthria and bilateral hand tremor and had also been diagnosed with WD at the same outside hospital.

On admission, his vital signs were normal. There was no jaundice, palmar erythema, or spider angioma. The liver was not palpable, whereas the spleen was palpable 2 cm below the left costal margin on deep inspiration. Neurological examination revealed an alert mental status, dysarthria, a broad-based gait, and mild limb hypotonia, with preserved muscle strength and deep tendon reflexes. Finger-to-nose and heel-to-shin testing showed mild bilateral dysmetria, whereas rapid alternating movements were intact. No Kayser–Fleischer (K-F) rings were observed on routine examination. Pyramidal and meningeal signs were absent. A total Leipzig score was 4.

**Figure 1.**
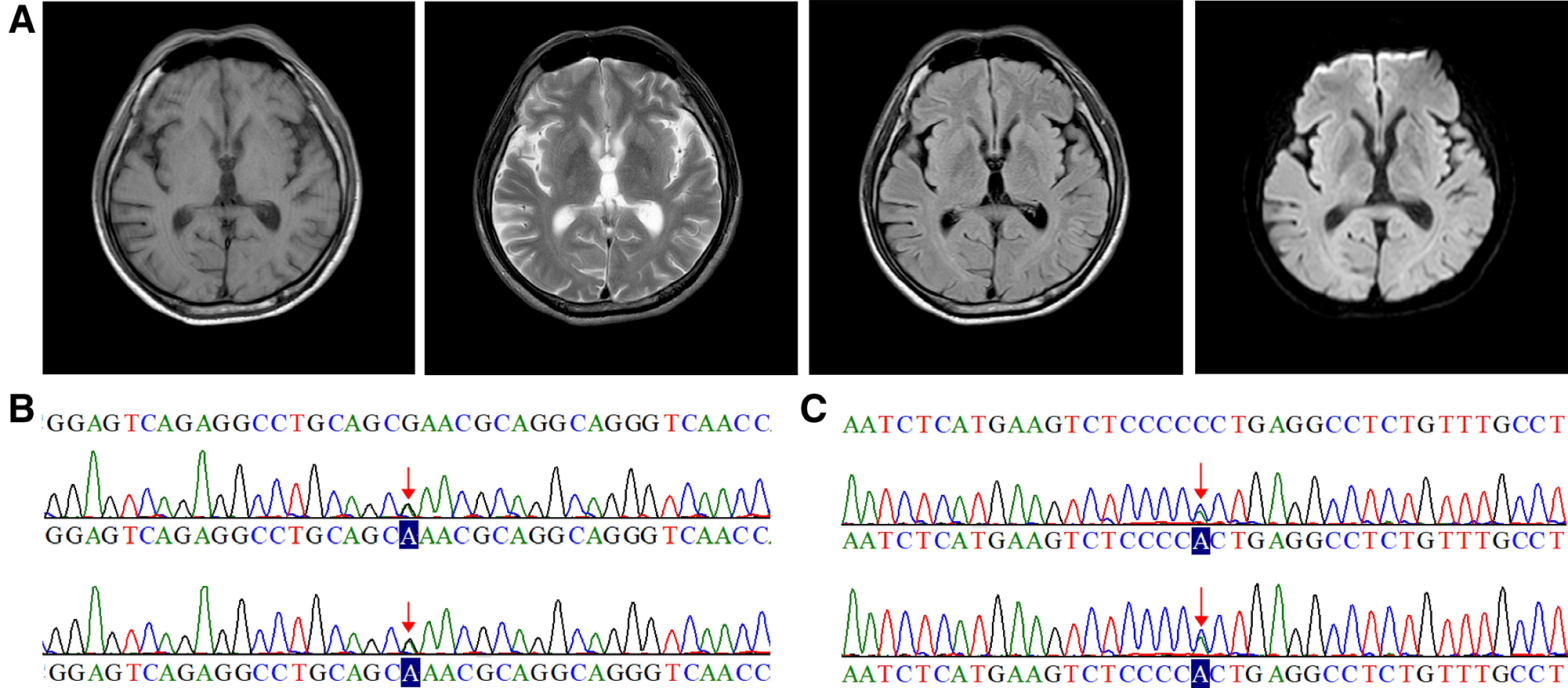
Brain MRI and *NPC1* gene sequencing results. (A) Brain MRI with diffusion-weighted imaging showed no obvious parenchymal abnormalities. (B) Sequencing results of the *NPC1* gene in the patient and his sister revealed 2 missense variants. Both individuals harbored a C > T substitution at position c.2932 in exon 20 of the *NPC1* gene (c.2932C > T), which resulted in an arginine-to-cysteine substitution at amino acid 978 (p.Arg978Cys, indicated by the arrow). (C) The patient also carried a G > T substitution at position c.2974 of the *NPC1* gene (c.2974G > T), leading to a glycine-to-tryptophan substitution at amino acid 992 (p.Gly992Trp, indicated by the arrow). MRI = magnetic resonance imaging.

**Figure 2.**
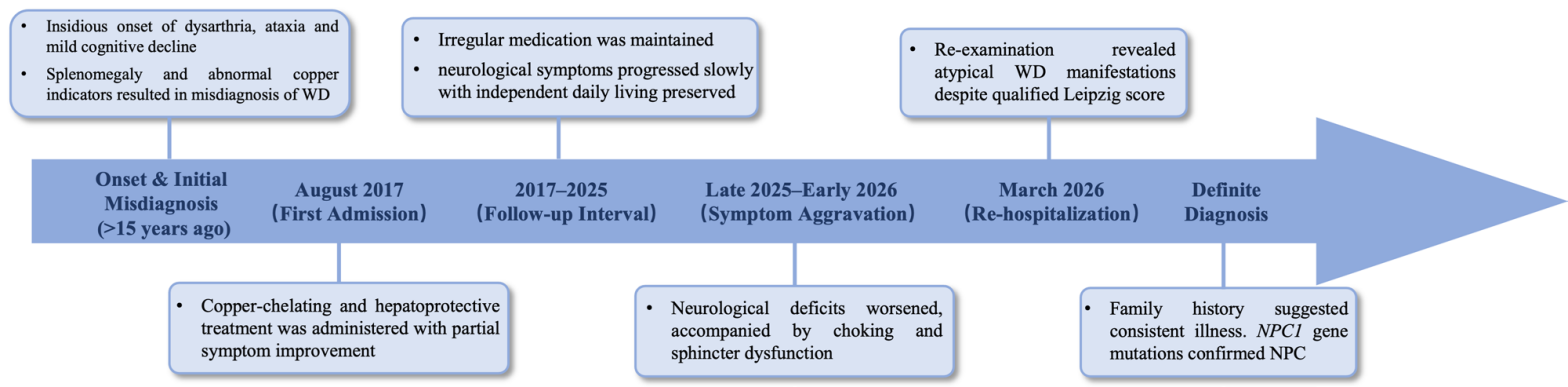
Patient clinical course timeline. WD = Wilson disease.

Laboratory testing showed leukopenia, with a white blood cell count of 2.87 × 10^9^/L, low serum ceruloplasmin of 0.125 g/L (normal reference range: 0.23–0.44 g/L), and elevated 24-hour urinary copper excretion of 396.60 µg/24 h (normal reference range: <100 µg/24 h). Urinalysis, stool examination, liver and renal function tests, fasting glucose, lipid profile, coagulation profile, thyroid function, and autoimmune screening were unremarkable. Abdominal ultrasonography showed heterogeneous hepatic echogenicity and splenomegaly measuring 177 mm × 54 mm. Chest computed tomography revealed bilateral pulmonary inflammatory changes. Brain magnetic resonance imaging (MRI) with diffusion-weighted imaging showed no evident parenchymal abnormalities (Fig. 1A). Although the biochemical abnormalities were compatible with WD, the overall presentation was atypical. Specifically, K-F rings were absent, brain MRI did not show characteristic WD-related changes, and the neurological phenotype was dominated by cerebellar ataxia. The remarkably slow progression despite long-term irregular anti-copper therapy further argued against typical WD. An alternative inherited disorder, including hereditary ataxia or NPC, was therefore considered. Genetic testing of the proband and his affected sister identified compound heterozygous pathogenic *NPC1* variants, c.2932C > T (p.Arg978Cys) (Fig. 1B) and c.2974G > T (p.Gly992Trp) (Fig. 1C), establishing the diagnosis of NPC. The clinical timeline is detailed in Figure 2.

## 3. Discussion

NPC is an autosomal recessive lysosomal lipid storage disorder caused by pathogenic variants in *NPC1* or *NPC2*, resulting in impaired intracellular trafficking of unesterified cholesterol and multiple sphingolipids. Its clinical presentation is highly heterogeneous, with variable involvement of the liver, spleen, and central nervous system. In adolescent- and adult-onset disease, progressive ataxia, dystonia, tremor, dysarthria, cognitive decline, and psychiatric or behavioral disturbances are common neurological manifestations; splenomegaly and vertical supranuclear gaze palsy may also occur.^[1,3,4]^ WD caused by pathogenic variants in *ATP7B* is an autosomal recessive disorder of copper metabolism with a wide clinical spectrum affecting the liver, brain, kidneys, and other organs.^[5]^ Despite their distinct molecular and pathophysiological bases, NPC and WD share several clinical and laboratory features, including overlapping age at onset, neurological presentations, splenomegaly, hematologic abnormalities, and biochemical abnormalities related to copper metabolism. Without genetic testing, these similarities may result in misdiagnosis. Our patient was initially diagnosed with WD and treated intermittently for presumed copper overload for several years. The unusually indolent disease course, absence of K-F rings, lack of characteristic brain MRI abnormalities, and predominance of cerebellar ataxia prompted diagnostic reconsideration. Genetic testing ultimately established the diagnosis of NPC. This case highlights 2 important diagnostic issues: the potential mechanisms underlying low ceruloplasmin and increased urinary copper excretion in NPC, and the limitations of the Leipzig scoring system when applied to patients with atypical or overlapping inherited metabolic disorders.^[6]^

Reduced serum ceruloplasmin and increased urinary copper excretion were major contributors to the initial misdiagnosis in this patient. However, hypoceruloplasminemia is not specific for WD.^[7]^ Aceruloplasminemia, a rare adult-onset autosomal recessive disorder within the spectrum of neurodegeneration with brain iron accumulation, is characterized by markedly reduced or absent ceruloplasmin. Owing to the ferroxidase activity of ceruloplasmin and its role in iron homeostasis, ceruloplasmin deficiency causes systemic iron overload, with iron deposition in the liver, retina, pancreas, and multiple brain regions, eventually leading to multiorgan damage.^[8,9]^ Menkes disease, or kinky hair disease, is another inherited disorder of copper metabolism. It is caused by pathogenic variants in *ATP7A*, resulting in impaired intestinal copper absorption and intracellular copper transport, copper deficiency, and consequently reduced ceruloplasmin levels.^[10,11]^ Therefore, reduced ceruloplasmin should not be interpreted as specific evidence of WD, particularly when the clinical phenotype or disease course is atypical. Recent studies have suggested that the reduced ceruloplasmin and increased urinary copper excretion observed in some patients with NPC may be related to impaired copper trafficking in late endosomes secondary to *NPC1* dysfunction. This defect may interfere with *ATP7B*-mediated incorporation of copper into ceruloplasmin and subsequent secretion of the holoenzyme, thereby resulting in reduced levels of functional ceruloplasmin. In addition, abnormal intracellular copper accumulation and impaired biliary copper excretion in the liver may further disturb systemic copper homeostasis, leading to low serum ceruloplasmin and elevated urinary copper excretion.^[12–14]^ Therefore, NPC may mimic WD not only clinically but also biochemically, making the differential diagnosis particularly challenging.

At present, the Leipzig scoring system remains the most widely used composite diagnostic tool for WD in Europe, the United States, and China. It incorporates several parameters, including K-F rings, neurological manifestations, serum ceruloplasmin, Coombs-negative hemolysis, hepatic copper quantification, 24-hour urinary copper excretion, and *ATP7B* variants, and assigns weighted scores to support the diagnosis of WD.^[15,16]^ Its main advantage lies in the integration of clinical, biochemical, and genetic data, which facilitates early screening and standardized diagnostic assessment. However, the Leipzig score has notable limitations in routine clinical practice.^[17]^ Several of its core components are not disease-specific, particularly low ceruloplasmin, elevated urinary copper excretion, and neurological manifestations. These findings may also be observed in NPC, chronic liver disease, cholestatic disorders, and other inherited metabolic conditions. Moreover, the Leipzig system is more useful for identifying abnormal copper metabolism than for establishing its underlying etiology. As a result, its specificity decreases considerably in atypical WD and in WD mimics. This limitation is particularly important in patients who present with neurological symptoms and only mild to moderate abnormalities in copper indices. In such cases, the diagnostic threshold may still be reached even when characteristic ophthalmologic findings or neuroimaging abnormalities are absent, thereby increasing the risk of misdiagnosis.

The present case illustrates this diagnostic pitfall. The patient had a total Leipzig score of 4, which met the clinical threshold for a diagnosis of WD, and was initially diagnosed as such at our center. Further evaluation, however, showed no characteristic brain MRI abnormalities typically seen in neurologic WD,^[5]^ and no K-F rings were identified. In light of the clinical phenotype, disease course, and subsequent genetic findings, the final diagnosis was revised to NPC. This case suggests that although the Leipzig scoring system is practical in typical WD, its diagnostic specificity is clearly limited in patients with atypical phenotypes, slow progression, neuroimaging findings inconsistent with WD, or features such as splenomegaly, prominent psychiatric or behavioral symptoms, and vertical supranuclear gaze palsy. In particular, when ceruloplasmin is only mildly reduced, urinary copper lacks a pretreatment baseline or has already been influenced by decoppering therapy, and there is no K-F ring or convincing evidence of liver disease, the score should not be overinterpreted, and further differential evaluation should be pursued.

## 4. Conclusion

In this case, decreased ceruloplasmin and elevated urinary copper led to a prolonged misdiagnosis of NPC as WD and unnecessary copper-chelating therapy until genetic testing confirmed the correct diagnosis. This highlights that key biochemical and clinical components of the Leipzig scoring system are not specific to WD, and that NPC may closely mimic WD in complex diagnostic settings. NPC should be considered in patients with progressive neurological deterioration, splenomegaly, psychiatric symptoms, or vertical supranuclear gaze palsy, particularly when K-F rings and typical WD-related neuroimaging findings are absent, even if the Leipzig score meets the diagnostic threshold. Early use of genetic testing and disease-specific biomarkers may improve diagnostic accuracy and prevent inappropriate treatment. Because prior long-term treatment may have altered the patient’s original phenotype, prospective studies are needed to further validate these observations.

## 5. Patient perspective

The patient stated, “For years, I believed I had Wilson disease and received intermittent treatment. After genetic testing confirmed Niemann–Pick disease type C, I felt relieved to finally understand my condition and hope my experience helps others receive an earlier diagnosis.”

## Acknowledgments

The authors gratefully acknowledge the technical support and collaboration provided by the colleagues in the Department of Neurology, the First Affiliated Hospital of Anhui University of Chinese Medicine.

## Author contributions

**Conceptualization:** Shang Xiang, Daiping Hua, Lijuan Zhang.

**Data curation:** Shang Xiang, Lanting Sun.

**Funding acquisition:** Wenming Yang, Han Wang.

**Project administration:** Han Wang.

**Writing – review & editing:** Han Wang.

**Writing – original draft:** Shang Xiang, Daiping Hua, Lijuan Zhang.
